# Effects of increasing axial load on cervical motor control

**DOI:** 10.1038/s41598-021-97786-3

**Published:** 2021-09-20

**Authors:** David Rafique, Ursula Heggli, Denis Bron, David Colameo, Petra Schweinhardt, Jaap Swanenburg

**Affiliations:** 1 Department of Chiropractic Medicine, Balgrist University Hospital, Zürich, Switzerland; 2 University of Zurich, Zürich, Switzerland; 3 AeMC, Aeromedical Center, Swiss Air Forces, Dubendorf, Switzerland; 4 Laboratory of Systems Neuroscience, Department of Health Science and Technology, Institute for Neuroscience, ETH, Zürich, Switzerland; 5 Integrative Spinal Research ISR, Department of Chiropractic Medicine, Balgrist University Hospital, Balgrist Campus, Lengghalde 5, 8008 Zürich, Switzerland

**Keywords:** Motor control, Somatosensory system

## Abstract

To investigate the effects of increasing axial load on cervical motor control. Surrogates of cervical motor control were active cervical range of motion (C-ROM) and joint position error (JPE) assessed in flexion, extension, lateroflexion and rotation directions in 49 healthy young men (mean age: 20.2 years). All measurements were executed with 0-, 1-, 2-, and 3-kg axial loads. Linear mixed models were used to assess the effects of axial loading and cervical movement-direction on C-ROM and JPE. Post-hoc analysis was performed to compare load levels. Axial loading (*p* = 0.045) and movement direction (*p* < 0.001) showed significant main effects on C-ROM as well as an interaction (*p* < 0.001). C-ROM significantly changed with 3-kg axial load by decreaseing extension (− 13.6%) and increasing lateroflexion (+ 9.9%). No significant main effect was observed of axial loading on JPE (*p* = 0.139). Cervical motor control is influenced by axial loading, which results in decreased C-ROM in extension and increased C-ROM lateroflexion direction.

## Introduction

A well-functioning cervical motor control (CMC) is essential to maintain balance during activities of daily living (ADL)^1^. Sensory inputs to CMC originate from proprioceptive, visual and vestibular systems^2,3^. Among these three systems, only proprioception directly interacts with mechanical axial loading^4^. Different methods are used to assess proprioception^5^. One reliable method to assess proprioception is the joint position error (JPE) test, a measure of the joint position sense^6^. The The cervical JPE measurement itself is a proxy for the afferent input from the cervical joints and cervical muscle receptors^7^. Therefore, JPE is used to detect any deviations of the CMC^7^. The JPE tests a subject’s ability to reposition, with eyes closed, a joint or a body part back to the original position after movement^8^. The JPE is mostly used to assess hip, knee, ankle, elbow and shoulder joints as well as spinal proprioception^8^. Proprioception itself is dependent on cervical flexibility^9^. When flexibility is limited, mechanoreceptor stimulation should be reduced, which in turn very likely results in decreased proprioception^10,11^. This cervical flexibility can be described by the active cervical range of motion (C-ROM)^10^. Previous studies assessing CMC by indirectly using active C-ROM and directly using the JPE did not include any external loading^5,12^. However, during ADLs, external loads such as wearing a helmet affect the muscles and joints^4^. Thus, testing both C-ROM and JPE with load would better mimic ADL. Moreover, external load has been shown to reduce JPE in the peripheral joints^13^. In contrast, effects of axial loading on JPE of the cervical spine are unknown. In vitro studies show an increased C-ROM during flexion and decreased during extension with loading^14^. However, the effect of axial loading on C-ROM in vivo and cervical JPE are currently unknown to our best knowledge. Unfortunately, axial loading has been reported as a risk factor for neck pain in aviators wearing headgear or in populations using their heads to carry loads (e.g. wood lifting or carrying water containers)^15–17^. In sports like American football, ice hockey and rugby, the incidence of neck injuries is high, primarily due to axial loading^18–21^. Furthermore, a recent study reported that additional axial loading of the cervical spine may lead to spinal structure overloading via the intervertebral discs and spine due to inadequate muscular and ligamentous stabilisation^22,23^. Moreover, neck pain itself is associated with decreased C-ROM^24^ and increased cervical JPE^25^. To our knowledge, relationships between axial loading and cervical proprioception have not yet been investigated. A possible CMC change caused by axial loading could have an impact on future preventive interventions, such as proprioceptive training under axial load.

The movements of the cervical spine are biomechanically and neurophysiologically complex^26^. The movements depend on the geometric parameters of the zygapophyseal joints, the intervertebral discs, and the uncovertebral joints^26^. Due to the geometric parameters, the movements are interdependent, and coupled movements occur^27^. One way to analyse coupled motions of the cervical spine are the use of three-dimensional (3-D) motion recordings. These allow accurate analysis of the movements and thus allow coupled motion to be analyzed^26^.

Thus, this study aimed to investigate the effects of increasing axial load on CMC by measuring active C-ROM and cervical JPE as alternative measures of proprioception.

## Results

A total of 50 male participants were recruited. One participant stopped measurements due to lack of motivation. 49 complete datasets were analysed. The mean age of included participants was 20.2 (± 1.4) years; weight, 73.7 (± 3.1) kg; height, 180.3 (± 1.8) cm; and NDI, 4.7 (± 1.4) %. Two incomplete datasets from the ROM measures were removed. No adverse events occurred during measurements. Four participants reported very mild neck pain, one participant light pain from the massage ball during the measurements. For technical reasons, 1% of the ROM and 2% of the JPE values are missing. Absolute C-ROM and JPE values are presented in Table 1. For C-ROM, maximum likelihood testing (MLT) suggested a full multiplicative linear mixed models (LMM) with interaction between axial loading and movement direction [*p* < 0.001, *df* = 18, χ^2^(9) = 58.91]. In contrast, for JPE, a reduced additive LMM without interaction between axial loading and movement direction [*p* = 0.898, *df* = 18, χ^2^(9) = 4.20] was suggested by MLT.

### Cervical range of motion (C-ROM)

**Figure 1:**
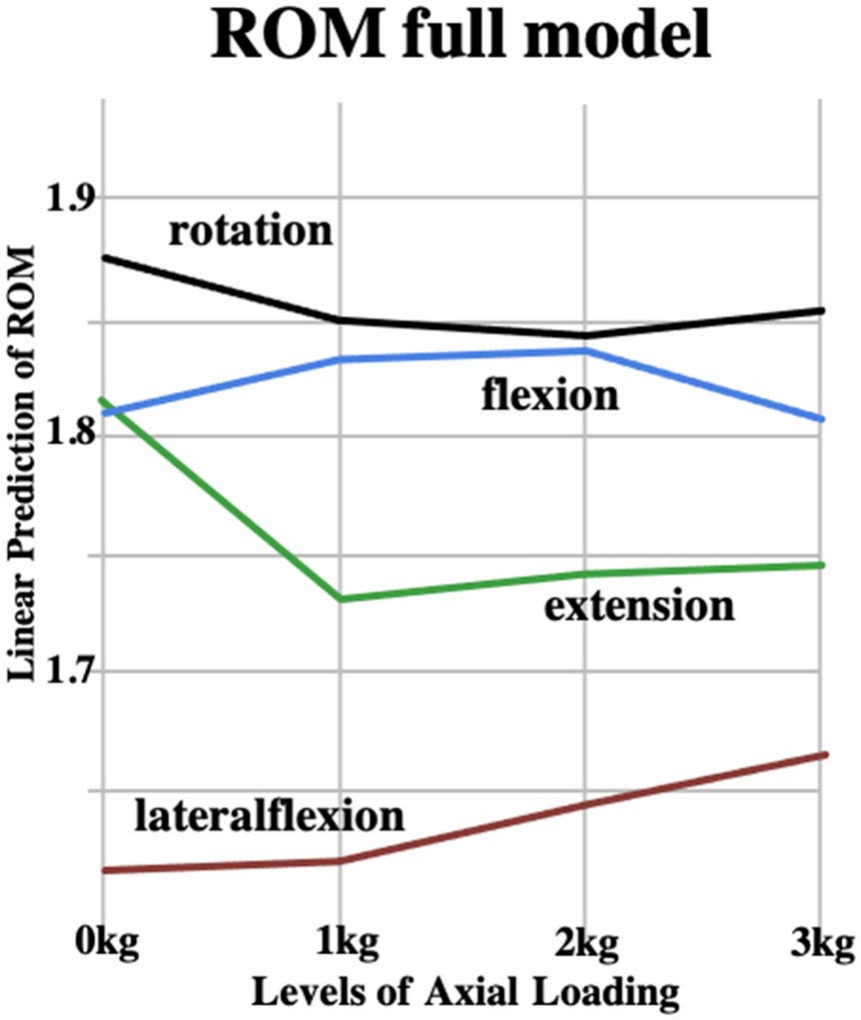
Linear prediction of the C-ROM full multiplicative model with interaction.

The full model indicated significant main effects; i.e. of axial loading (*p* < 0.043, *df* = 3, F = 2.733) and of movement direction (*p* < 0.001, *df* = 3, F = 405.994). Moreover, a significant interaction was observed between axial loading and movement direction (*p* < 0.001, *df* = 9, F = 6.682). Post-hoc analysis indicated that C-ROM decreased by 13.6% in extension (*p* < 0.001) and increased by 9.9% in lateroflexion (*p* < 0.011) from 0 to 3-kg axial loading (Tables 1 and 2 and Fig. 1). No axial loading changes were observed in the flexion and rotation directions.

### Joint position error (JPE)

**Figure 2:**
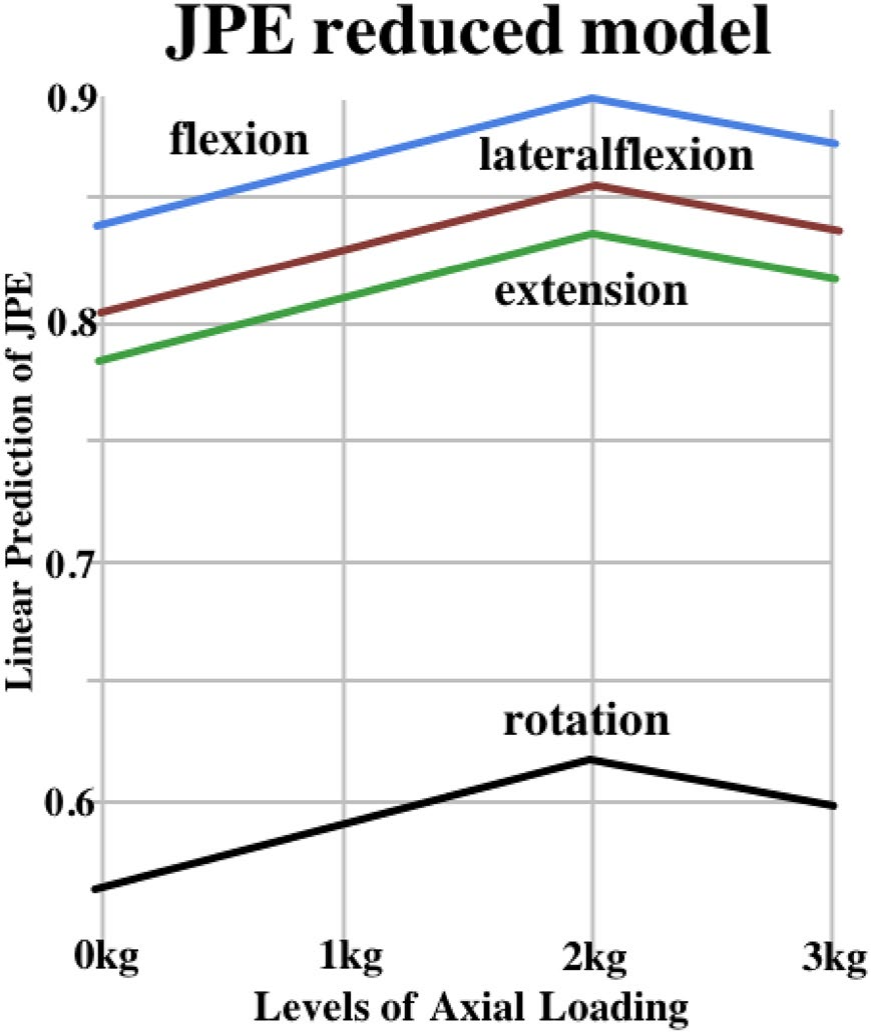
Linear prediction of the JPE reduced additive model without interaction.

Movement direction (*p* < 0.001, df = 3, F = 50.391) showed a significant main effect on JPE. An analysis revealed no significant main effect on JPE with axial loading (*p* = 0.139, *df* = 3, F = 1.835). A reduced model was created, where the effect on JPE was significant for the 2-kg axial loading. JPE increased by 12.1% (*p* = 0.034, *df* = 720.89) as compared to the no-loading condition (Tables 1 and 3 and Fig. 2). No changes were observed for 1- and 3-kg loading levels. Reduced LMM did not allow distinguishing axial loading effects for individual movement directions, as no interaction effect was observed. Additional plots and tables with more detailed information on the results are shown in Supplementary File 1 and 2.

## Discussion

Axial loading resulted in decreased C-ROM in the extension direction but increased in lateroflexion. A previous in vitro study showed partly similar results, namely, a decrease in extension and an increase in lateroflexion^14^. However, it showed an increase in flexion and rotation with axial load, a finding inconsistent with that of the present study. This discrepancy in findings might be that larger loads were used in the in vitro study, specifically 10–150 times larger than those in the present study^14^. Another possible explanation for decreased C-ROM in the extension direction with axial loading could be the conscious and nonconscious attempts to protect the cervical myelon^28^. A previous study demonstrated changes in the spinal canal diameter during cervical flexion and extension with reduced sagittal diameter being greater in extension than in flexion^29^. Therefore, additional axial loading could enhance this effect, and supraspinal protective reflexes would inhibit the extension via Ib inhibitory sensory afferents^10^.

In contrast to the effects of axial loading on cervical extension, lateroflexion increased with additional axial loading. Passive stretching of the contralateral neck muscles may have led to increased lateral flexion. This contralateral stretching in lateroflexion was provoked by the additional 3-kg axial load. Cervical lateroflexion and rotation are known to biomechanically coupled in the same direction^30^; therefore, such an effect would also be expected in the rotation direction. Nevertheless, no C-ROM changes were found in the rotation direction by axial loading. We assume that the isolated lateroflexion might represent an unnatural movement of the cervical spine (20), is therefore less controlled, and thereby allows passive contralateral stretch of the neck muscles under an additional axial load. Measuring and considering these two movements separately in vivo have been shown to be difficult^30^. Furthermore, another study reported that isolated rotational movement in the middle and lower cervical spine was described as biomechanically possible, whereas isolated lateroflexion is blocked by aligning the intervertebral joints^21^. Nevertheless, isolated lateroflexion performed in the current study could explain this above-mentioned deviation from the coupling behaviour.

JPE was found to be increased with load irrespective of movement direction, albeit the increase reached significance with 2-kg axial loading. JPE results in this study might be explained by the sensory-perceptual system by Shumway–Cook^10^. Sensory-perceptual systems can be further divided into vestibular, visual and somatosensory systems. Vestibular function is affected by the axial load only within the scope of acceleration^31^. In the present study, acceleration can be neglected because no dynamic variables were measured. The role of the visual input system can be similarly neglected because all measurements were performed with eyes closed. In contrast, proprioception might be affected by the additional weight because cervical anatomical structures (ligaments, joint capsules and muscles) contain a large number of proprioceptors^32^ that might be disrupted and overstimulated by the additional axial load, which could impair cervical proprioception by miscalculating posture and cervical position^32^. We hypothesise that miscalculations due to new and unknown sensory input contributed to the observed increase in JPE.

Experimentally overloaded action systems might have contributed to the observed reduced CMC during axial loading^10,33^. Overloading refers to a perturbation of highly automated movement, such as reaching out with an arm. A new situation or stimulus, such as additional axial load, creates an overload and provokes inappropriate muscle contractions in ADL^10,33^. This new stimulus requires the learning of an adequate motor response, which is first registered in the supplementary motor and premotor areas of the CNS. Then, these motor programmes have to be trained and learned to promote adequate motor responses to a specific stimulus in proprioceptors^10^. For example, experienced high-performance military aviators previously exposed to high G-forces showed lower neck muscle activity as compared to beginners^34^. An inexperienced person enduring additional cervical axial load may have greater cervical proprioception disturbances than an experienced person. Therefore, the tendency of increased JPE may be a consequence of non-adaptation to the new, unknown loading situation.

Comparing JPE findings with the existing literature in this study is difficult because of different measurement methods. Previously, two-dimensional (2D) methods such as laser projections on the wall have been used^5,32^. This study used a 3D movement tracking method, showing higher JPE (e.g. 13.9% in flexion) than 2D methods^10,30^. One previous study was found to have also used a 3D movement tracking system to measure C-ROM and JPE^35^. JPE values of that study were similar to that of the present study^9^. A possible explanation for the discrepancy between 2 and 3D methods might be that speed and direction changes in the third dimension are not included in 2D methods.

Some methodological limitations should be mentioned. During the data collection procedure, fixation of the thoracal and lumbar spine with a massage ball at Th2–Th4 levels might not be fully sufficient, which could potentially have led to larger C-ROM values. Therefore, the absolute C-ROM values should be interpreted with caution. Furthermore, the order of movement-direction measurements (lateroflexion left-lateroflexion right-flexion-extension-rotation left-rotation right) could have led to a certain learning effect for JPE. Calibration was only done when the participant could not centre the point on the screen by themself. This could have led to possible bias.

Future studies should include a precise standardization regarding the timing and amount of calibrations. Reliability and validity of the 3D cervical trainer used in this study are warranted. Comparable electromagnetic or ultrasound-based 3D measurement devices have been proven to be mature and reliable^11,36^. Another limitation of this study is that only asymptomatic young males were included. Because age influences JPE^37^, the generalisability of the results of the present study is not known. An important aspect that was not addressed in this study is the effect of time on JPE, as the helmet usually has to be worn for many hours. This should be addressed in future studies. In addition, the daily use of a helmet could have influenced the outcome and led to an adaptation of the CMC. In this case, the effect of daily helmet use would have weakened the effect of additional load, which might be even larger in individuals who are not used to wearing helmets There were 1 to 2% of the ROM and JPE measures missing. There is a small chance that these missing values caused a bias in the results; however, it is highly unlikely that this would influence their interpretation.

Cervical axial loading seems to trigger protective active and passive mechanisms, possibly causing decreased cervical extension-ROM and increased cervical lateroflexion-ROM. A 2-kg axial loading might lead to increased cervical JPE. Therefore, additional cervical loading seems to be a disturbing factor for CMC. Integrating higher axial loading into rehabilitation or prevention exercises might be useful, especially if an individual should perform heavy physical work or endure axial spinal loading, such as personnel wearing headgears like rescue personnel, soldiers or high-performance military aviators or in populations using their heads to carry loads.

## Methods

### Participants

A total of 50 asymptomatic young adult men aged 18–24 years, who were Swiss military employees, were recruited. Potential participants with any current or chronic spinal pain, aged < 18 years, and with a neck disability index (NDI) questionnaire percentage score of ≥ 15 were excluded^38^. Swiss military employees constitute a representative population, as they are mostly men and wear helmets most of the time and are consequently exposed to additional cervical axial loads. Five NDI measures were invalid because five participants misinterpreted the NDI questionnaire. Other assessment showed no neck pain, these participants were clear to participate. The study was approved by the local ethics committee of the Canton of Zurich (BASEC 2019-00830). Methods were carried out in accordance with relevant guidelines and regulations.

All participants provided written informed consent before inclusion in the study in accordance with the Code of Ethics of the World Medical Association (Declaration of Helsinki). The study was registered ClinicalTrials.gov Identifier: NCT04434235, 01.10.2019).

### Data collection procedures

The study procedure was explained to all study participants. Active C-ROM was measured first, followed by cervical JPE measurements. Six C-ROM directions and JPE were measured in the following order: flexion, extension, lateroflexion left, lateroflexion right, rotation left and rotation right. The order of measurement directions was not randomised. The selected load levels were 0, 1,2, and 3 kg. These loads are comparable with that of different helmets like motor cycle helmets (≈ 1.5 kg), army helmets (≈ 1 kg) and helmets of military jet and helicopter aircrews (≈ 2.5 kg). Axial loading (0, 1, 2 and 3 kg) was randomly added, starting or ending with 0 kg. Active C-ROM was measured based on the maximum motion assisted by the examiner. The participants were asked to move the head as far as possible in all directions. For the JPE, participants made a red-marked measurement point at the centre provided by the software. The purpose of the screen was to provide visual feedback to participants so that they could re-centre the dot before starting the next JPE measurement. Participants were then asked to close their eyes, turn their head at their own speed as far as they could in that direction, and then return to the starting position. Once the participant indicated that they had returned to the starting position, the measurement was stopped. The difference between start and end positions was defined as the JPE^8^. After each JPE measurement, the participant could open his eyes and re-centre the JPE measurement point. Calibration of the centre point was required if the participant could not centre the point on the screen by himself, because the third dimension could not be represented on the screen. Then, calibration allowed further measurements starting from a straight position that was comfortable for the participant. ROM was measured once in each movement direction and loading level. JPE measurements were repeated three times before measuring the next direction or axial loading level. The average of these three measurements was used for analysis. In addition, left and right directions (for lateroflexion and rotation) were combined to reduce the number of movement-direction levels, especially because no significant differences between left and right direction measurements were expected in group measurements^11^. All participants were asked to report any adverse events, e.g. pain during the measurements, immediately.

### Measurement setup

**Figure 3:**
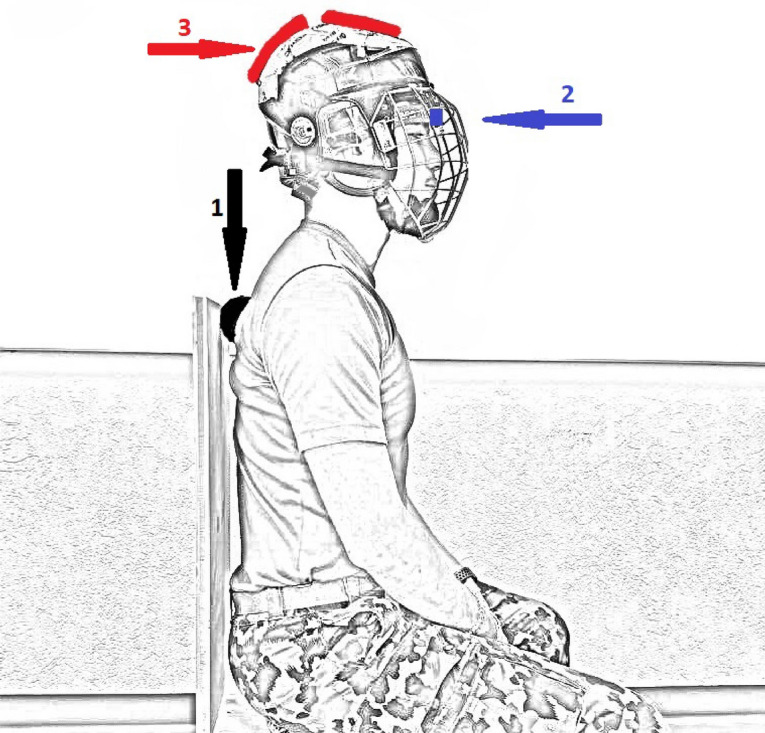
Participant measurement setup with 2 kg axial loading. Figure 3 shows the participant measurement setup here with 2-kg axial load (1 kg = [helmet + first weight] and [second weight] = 1 kg). For 1 kg axial loading, the second weight was removed and for 3 kg axial loading a third (1 kg-) weight was attached. Arrow 1: Massage ball placed at Th2–Th4 (The thoracic vertebra), Arrow 2: Sensamove device (point source) as a front-band, Arrow 3: A 2-kg axial load manually balanced over the sagittal balance axis (around the meatus acusticus externus).

Participants sat on a costume-built chair (120 × 42 × 42 cm, sitting the table height at 42 cm) for the experiment, with a straight flat woodboard to hold their backs in a stable position. A 500 small massage ball (Antonia, Decathlon) was placed at the thoracic vertebra (Th2–Th4) to reduce thoracic and lumbar movements. The upper body was fixed by activating the trunk muscles to hold the massage ball in position to ensure that only segments proximal to Th2–Th4 could be actively moved. Additional loads were attached to an ice hockey helmet (CCM, M-size) with Velcro®. Loads were adjusted so that the helmet’s total weight was 1, 2 or 3 kg and were manually balanced around the sagittal balance axis (the meatus acusticus externus) on the helmet. Helmet slippage was controlled by securing the fixation strap to the helmet. C-ROM and JPE were measured using a 3D Cervical Trainer device (Sensamove, Groessen, The Netherlands) connected via Bluetooth to a laptop with 3D Cervical Trainer software (www.sensamove.com). The measurement setup is displayed in Fig. 3.

### Statistical analysis

Descriptive statistics were used for participant characteristics. The Shapiro–Wilk test was used to test for normality. C-ROM and JPE data were not normally distributed and were therefore log10-transformed. Sphericity was visually assessed using scatter plots of raw C-ROM and JPE values.

LMMs were used for ROM and JPE. Models were constructed separately for two dependent variables, C-ROM and JPE. Independent variables were cervical axial loading (four levels: 0, 1, 2 and 3 kg) and movement direction (four levels: flexion, extension, lateroflexion and rotation). For each independent variable (C-ROM and JPE), two models were constructed with and without interaction terms between two independent variables and compared using the MLT. Interaction models for ROM and without interaction for JPE were selected as final models. Participant ID was included as a random effect factor. Post-hoc analysis was performed for C-ROM and JPE. More detailed information on MLT and model selection is shown in Supplementary File and Tables S1–S3 or Supplementary File 2 . ,IBM SPSS Statistics 23 for Windows (Inc; Chicago, Illinois) and R version 3.6.2 software (R Foundation for Statistical Computing, Vienna, Austria) were used for all statistical analyses. R packages *lme4*, *lmerTest* and *emmeans* for post-hoc multiple testing provided all necessary modelling tools.

### Ethics approval and consent to participate

The study was approved by the local ethics committee of the Canton of Zurich (BASEC 2019–00830). and registered ClinicalTrials.gov Identifier: NCT04434235, 01.10.2019). All participants provided written informed consent before inclusion in the study.

**Table 1:** Absolute means of the ROM and JPE values in degrees by 0-, 1-, 2- and 3-kg axial loading.

	Flexion (95% CI lower–upper) in degrees [°]	SD	Extension (95% CI lower–upper) in degrees [°]	SD	Lateroflexion (95% CI lower–upper) in degrees [°]	SD	Rotation (95% CI lower–upper) in degrees [°]	SD
**C-ROM** **(n = 47)**								
0 kg	64.88 (± 3.46)	12.47	65.14 (± 3.09)	11.14	42.48 (± 2.19)	7.89	75.12 (± 2.73)	9.85
1 kg	69.44 (± 3.10)	11.20	56.06 (± 3.65)*	13.16	43.03 (± 2.35)	8.48	71.14 (± 4.53)	16.34
2 kg	68.98 (± 3.74)	13.51	56.64 (± 3.59)*	12.95	44.52 (± 2.39)	8.62	71.36 (± 3.86)	13.91
3 kg	65.52 (± 3.58)	12.92	57.12 (± 3.45)*	12.44	46.92 (± 2.25)*	**8.11**	72.36 (± 3.44)	12.41
**JPE** **(n = 49)**								
0 kg	8.62 (± 2.33)	8.41	7.99 (± 2.00)	7.20	7.84 (± 1.64)	5.93	4.20 (± 0.56)	2.01
1 kg	10.18 (± 2.43)	8.75	8.80 (± 2.15)	7.74	7.78 (± 1.80)	6.50	4.16 (± 0.51)	1.85
2 kg*	**10.80 (± 2.42)**	**8.70**	**9.48 (± 2.14)**	**7.71**	**8.67 (± 1.78)**	**6.41**	**4.72 (± 0.78)**	**2.82**
3 kg	9.02 (± 1.53)	5.53	7.79 (± 1.33)	4.80	8.20 (± 1.32)	4.78	4.18 (± 0.58)	2.08

C-ROM cervical range of motion, JPE joint position error, 95%-CI 95% confidence interval, SD standard deviation, kg kilogram, ° degrees, * significant (p < 0.05).

**Table 2:** Post-Hoc Analysis Table for C-ROM.

	Estimate [°], log10 transformed data	Sign. C-ROM change (10^Estimate)-1	SE	t value	*p* value
**Flexion**					
0–1 kg	0.0256		0.0133	− 1.9184	0.2221
0–2 kg	0.0286		0.0133	− 2.1428	0.1407
0–3 kg	− 0.0005		0.0133	0.0355	0.9999
**Extension**					
0–1 kg	− 0.0783	**(−) 16.5%**	0.0133	5.8766	** < 0.0001**
0–2 kg	− 0.0673	**(−) 14.3%**	0.0133	5.0447	** < 0.0001**
0–3 kg	− 0.0637	**(−) 13.6%**	0.0133	4.7793	** < 0.0001**
**Lateroflexion**					
0–1 kg	0.0020		0.0133	− 0.1489	0.9988
0–2 kg	0.0213		0.0133	− 1.5981	0.3804
0–3 kg	0.0412	**( +) 10.0%**	0.0133	− 3.0909	**0.0111**
**Rotation**					
0–1 kg	− 0.0242		0.0133	1.8132	0.2679
0–2 kg	− 0.0298		0.0133	2.2354	0.1148
0–3 kg	− 0.0203		0.0133	1.5265	0.4221

*C-ROM cervical* range of motion, *Estimate* effect size, *SE* standard error, *NA* not applicable, *kg* kilogram, +  = increase,—= decrease, % percent.

This table shows the post-hoc analysiy of the C-ROM model with the log10-transformed estimates in degrees[°] (fixed effects), significant C-ROM changes in percent with C-ROM-changes in percent [%], standard error and significance levels.

**Table 3:** Table of fixed effects for JPE.

	Estimate [°] log10-transformed data	Sign. JPE-fold change [1- (10^Estimate) 1] * 100 [%]	SE	t value	*p* value
Flexion	0.0531	**(+) 12.3%**	0.0236	2.253	**0.0324**
Extension (intercept)	0.7879		0.0292	27.028	< 0.001
Lateroflexion	0.0167		0.0237	0.706	0.4804
Rotation	− 0.2202	**(−) 39.8%**	0.0236	− 9.335	** < 0.001**
Load 1 kg	0.0264		0.0235	1.121	0.2626
Load 2 kg *	0.0505	**(+) 12.3%**	0.0235	2.143	**0.0324**
Load 3 kg	0.0316		0.0235	1.344	0.1794

This table shows the fixed effects on JPE of the independent variables of cervical load and direction of movement fit through a linear mixed model with the log10-transformed estimates in degrees[°] (fixed effects), significant JPE changes and JPE-changes in percent [%], standard error and significance levels.

JPE joint position error, Estimate effect size, SE standard error, NA not applicable, kg kilogram, +  = increase,− = decrease, % percent.

## Supplementary Information

Supplementary Information 1.

Supplementary Information 2.

## Abbreviations

CNS: Central nervous system
CMC: Cervical motor control
C-ROM: Cervical range of motion
JPE: Joint position error
LMM: Linear mixed model
MLT: Maximum likelihood testing
NDI: Neck disability index
Th: Thoracic vertebra
