# Supplementary material for: Effects of increasing axial load on cervical motor control
Source: Sci Rep. 2021 Sep 20;11:18627. doi: 10.1038/s41598-021-97786-3 (PMC8452641; doi:10.1038/s41598-021-97786-3)
Supplement: Supplementary file 1 — Supplementary Information 1. [file 41598_2021_97786_MOESM1_ESM.docx]

**Effects of Increasing Axial Load on Cervical Motor Control**

David Rafique, Ursula Heggli, Denis Bron, David Colameo, Petra Schweinhardt, Jaap Swanenburg

**Supplementary File S1**

**Table S1**

Maximum likelihood testing (MLT): ANOVA of the reduced model (without interaction) and full model (with interaction) for both ROM and JPE

> anova(full.model, red.model)

| **ROM** | Df | AIC | BIC | deviance | Chisq | P (>Chisq) |
| --- | --- | --- | --- | --- | --- | --- |
| red.model |  | -1813.3 | -1771.7 | -1831.3 | - | - |
| **full.model** | **9** | **-1855.0** | **-1771.8** | **-1891.0** | **59.714** | **<0.001** |
|  |  |  |  |  |  |  |
| **JPE** |  |  |  |  |  |  |
| **red.model** |  | **33.695** | **75.582** | **15.695** | - | - |
| full.model | 9 | 49.211 | 132.985 | 13.211 | 2.4843 | 0.9813 |

*ROM* range of motion, *JPE* joint position error, *Df* degrees of freedom, *AIC* akaike information criterion, *BIC* bayesian information criterion, *Chisq* chisquare, *P* p-value, *red.model* reduced model (log10value~Load+Type+(1|Proband))

*Full.model* full model (log10value~Load*Type+(1|Proband))

**Table S2**

Type III ANOVAs (Satterthwaite’s method) of the reduced model and full model for **ROM**.

| **red.model**  > anova(red.model) | Sum Sq | Mean Sq | NumDF | F value | Pr (>F) |
| --- | --- | --- | --- | --- | --- |
| Load | 0.0338 | 0.01127 | 3 | 2.5113 | 0.05759 |
| Type | 5.0779 | 1.69265 | 3 | 377.2672 | <0.001 |
|  |  |  |  |  |  |
| **full.model**  > anova(full.model) |  |  |  |  |  |
| Load | 0.0338 | 0.01127 | 3 | 2.6981 | 0.04493 |
| Type | 5.0779 | 1.69265 | 3 | 405.3276 | <0.001 |
| Load:Type (interaction) | 0.2547 | 0.02830 | 9 | 6.7767 | <0.001 |

*ROM* range of motion, *Load* axial loading, *Type* movement direction, *Load:Type* interaction between load and type, Sum *Sq* sums of squares, *Mean Sq* means of squares, *NumDf* number degrees of freedom*,Pr* p-value, * significant effect

*red.model* reduced model (log10value~Load+Type+(1|Proband)

*Full.model* full model (log10value~Load*Type+(1|Proband)

**Table S3**

Type III ANOVAs (Satterthwaite’s method) of the reduced model and full model for **JPE.**

| **red.model**  > anova(red.model) | Sum Sq | Mean Sq | NumDF | F value | Pr (>F) |
| --- | --- | --- | --- | --- | --- |
| Load | 0.2523 | 0.08411 | 3 | 1.5651 | 0.1965 |
| Type | 8.9705 | 2.99015 | 3 | 55.6420 | <0.001 |
|  |  |  |  |  |  |
| **full.model**  > anova(full.model) |  |  |  |  |  |
| Load | 0.2517 | 0.08388 | 3 | 1.5467 | 0.2011 |
| Type | 8.9702 | 2.99006 | 3 | 55.1337 | <0.001 |
| Load:Type | 0.1322 | 0.01469 | 9 | 0.2708 | 0.9823 |

*JPE* joint position error, *Load* axial loading, *Type* movement direction, *Load:Type* interaction between load and type, Sum *Sq* sums of squares, *Mean Sq* means of squares, *NumDf* number degrees of freedom*,Pr* p-value, * significant effect

*red.model* reduced model (log10value~Load+Type+(1|Proband)

*Full.model* full model (log10value~Load*Type+(1|Proband)

**Figure S1**

Absolut ROM values, visualization.


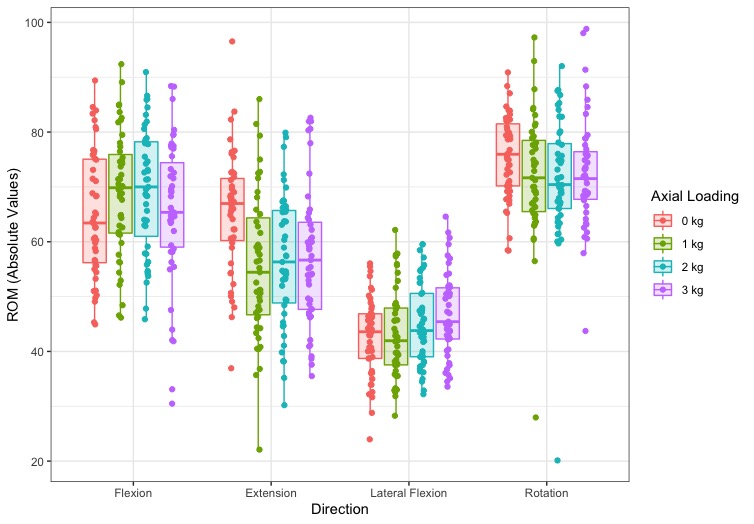


*ROM* range of motion, *kg* kilogram

**Figure S2**

ROM log transformed


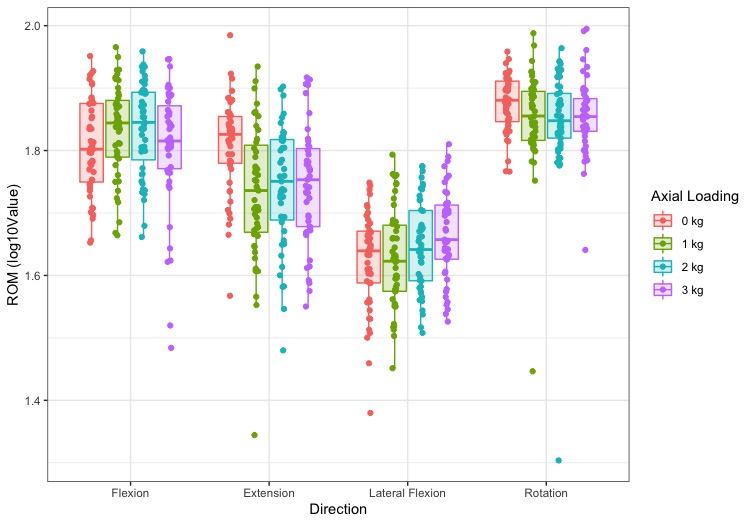


*ROM* range of motion, *kg* kilogram

**Figure S3**

Absolut JPE values, visualization.


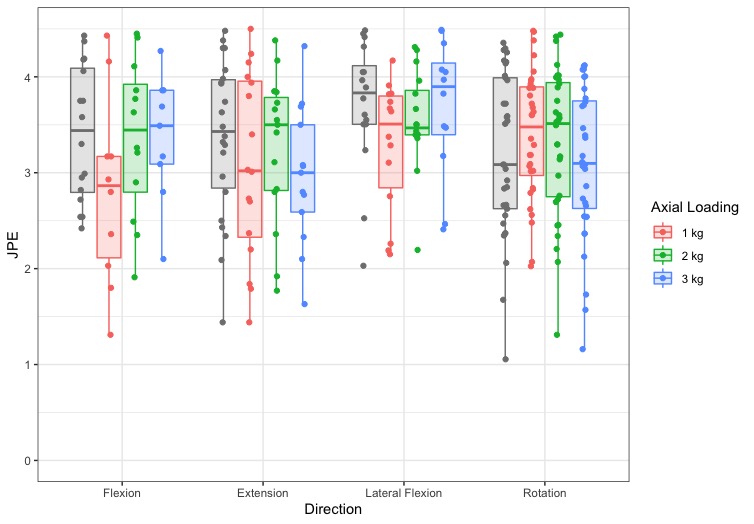


*JPE* joint position error, *kg* kilogram

**Figure S4**

JPE log transformed


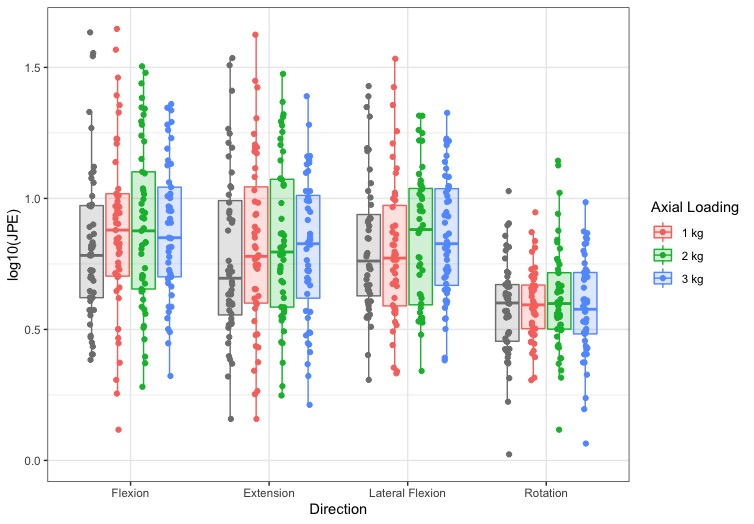


*JPE* joint position error, *kg* kilogram
