# Supplementary material for: Effects of increasing axial load on cervical motor control
Source: Sci Rep. 2021 Sep 20;11:18627. doi: 10.1038/s41598-021-97786-3 (PMC8452641; doi:10.1038/s41598-021-97786-3)
Supplement: Supplementary file 2 — Supplementary Information 2. [file 41598_2021_97786_MOESM2_ESM.html]

Effects of Increasing Axial Load on Cervical Motor Control


# Effects of Increasing Axial Load on Cervical Motor Control

### Statistical Analysis

#### David Colameo

#### 06/08/2021

### Load Dependencies

```
library(readxl)
library(lmerTest)
library(lme4)
library(reshape2)
library(emmeans)
library(ggplot2)
library(DT)
library(dplyr)
library(ggpubr)
library(rstatix)
```

# Range of Motion (ROM)

## Load Data and Prepare Tables

Load Raw data and log10-transform them

```
data <- data.frame(read_excel("./ROM-Axial Load on Cervical Spine.xlsx"))

data$Proband <- as.factor(data$Proband)
data[,c(2,3,4,5)] <- sapply(data[,c(2,3,4,5)], FUN=as.numeric)

#take out probands who had missing measurements (row-wise) with NaNs
data <- data[complete.cases(data),]
data <- melt(data, id.vars=c("Proband", "Type"), variable.name = "Load")
data <- aggregate(data, by=list(Type=data$Type, Proband=data$Proband, Load=data$Load), FUN=mean, na.rm=TRUE)
data <- data[, colSums(is.na(data)) != nrow(data)]


## Log-Transform Data
data$log10Value <- log10(data$value)


DT::datatable(data)
```

## Summarize Table

```
dat <- data %>%
    group_by(Load, Type) %>%
    summarize(count = n(),
              Mean = mean(value, na.rm=TRUE),
              Standard.Deviation = sd(value, na.rm=TRUE),
              lower.CI = mean(value, na.rm=TRUE) - qt(1 - (0.05 / 2), n() - 1) * sd(value, na.rm=TRUE) / sqrt(n()),
              upper.CI = mean(value, na.rm=TRUE) + qt(1 - (0.05 / 2), n() - 1) * sd(value, na.rm=TRUE) / sqrt(n())) %>% mutate_if(is.numeric, signif, digits=4)
```

```
## `summarise()` has grouped output by 'Load'. You can override using the `.groups` argument.
```

```
## `mutate_if()` ignored the following grouping variables:
## Column `Load`
```

```
DT::datatable(dat)
```

## Normality Testing

First with all values together: - create QQ-Plots, histograms and perform a Shapiro-Wilk Normality Test (p<0.05 is non-normally distributed)

```
p1 <- ggqqplot(data$value) + labs(title="QQ-Plot of all Raw Values")
p2 <- ggqqplot(data$log10Value) + labs(title="QQ-Plot of all log-transformed Values")

p3 <- ggdensity(data$value)+ labs(title="Histogram of all Raw Values")
p4 <- ggdensity(data$log10Value)+ labs(title="Histogram of log-transformed Values")

ggarrange(p1, p2, p3, p4,
          ncol = 2, nrow = 2)
```

```
## Raw Values
shapiro.test(data$value)
```

```
## 
##  Shapiro-Wilk normality test
## 
## data:  data$value
## W = 0.98479, p-value = 4.815e-07
```

```
## Log10-Transformed Values
shapiro.test(data$log10Value)
```

```
## 
##  Shapiro-Wilk normality test
## 
## data:  data$log10Value
## W = 0.96242, p-value = 5.706e-13
```

Now split according to groups (Load ~ Type) and plot raw values and perform Shapiro-Wilk Normality Tests (p<0.05 is non-normally distributed)

```
ggqqplot(data, "value", ggtheme = theme_bw()) +
  facet_grid(Load ~ Type, labeller = "label_both") + labs(title="QQ-Plots of group-splitted raw values")
```

```
ggdensity(data , "value") +
  facet_grid(Load ~ Type, labeller = "label_both") + labs(title="Histograms of group-splitted raw values")
```

```
data$rawVal <- as.numeric(data$value)
data$variable <- NULL
data %>%
  group_by(Load, Type) %>%
  shapiro_test(rawVal) %>%
 add_significance("p")
```

```
## # A tibble: 16 x 6
##    Type           Load  variable statistic         p p.signif
##    <chr>          <fct> <chr>        <dbl>     <dbl> <chr>   
##  1 extension      X0kg  rawVal       0.973 0.334     ns      
##  2 flexion        X0kg  rawVal       0.971 0.284     ns      
##  3 lateralflexion X0kg  rawVal       0.985 0.782     ns      
##  4 rotation       X0kg  rawVal       0.980 0.595     ns      
##  5 extension      X1kg  rawVal       0.984 0.759     ns      
##  6 flexion        X1kg  rawVal       0.987 0.864     ns      
##  7 lateralflexion X1kg  rawVal       0.959 0.0973    ns      
##  8 rotation       X1kg  rawVal       0.914 0.00202   **      
##  9 extension      X2kg  rawVal       0.984 0.759     ns      
## 10 flexion        X2kg  rawVal       0.975 0.405     ns      
## 11 lateralflexion X2kg  rawVal       0.957 0.0814    ns      
## 12 rotation       X2kg  rawVal       0.856 0.0000374 ****    
## 13 extension      X3kg  rawVal       0.954 0.0648    ns      
## 14 flexion        X3kg  rawVal       0.951 0.0462    *       
## 15 lateralflexion X3kg  rawVal       0.977 0.491     ns      
## 16 rotation       X3kg  rawVal       0.951 0.0454    *
```

Now split according to groups (Load ~ Type) and plot log-transformed values

```
ggqqplot(data, "log10Value", ggtheme = theme_bw()) +
  facet_grid(Load ~ Type, labeller = "label_both") + labs(title="QQ-Plots of group-splitted log-transformed values")
```

```
ggdensity(data , "log10Value") +
  facet_grid(Load ~ Type, labeller = "label_both") + labs(title="Histogram of group-splitted log-transformed values")
```

```
data %>%
  group_by(Load, Type) %>%
  shapiro_test(log10Value) %>%
 add_significance("p")
```

```
## # A tibble: 16 x 6
##    Type           Load  variable   statistic             p p.signif
##    <chr>          <fct> <chr>          <dbl>         <dbl> <chr>   
##  1 extension      X0kg  log10Value     0.948 0.0350        *       
##  2 flexion        X0kg  log10Value     0.976 0.453         ns      
##  3 lateralflexion X0kg  log10Value     0.954 0.0638        ns      
##  4 rotation       X0kg  log10Value     0.966 0.184         ns      
##  5 extension      X1kg  log10Value     0.959 0.0948        ns      
##  6 flexion        X1kg  log10Value     0.970 0.276         ns      
##  7 lateralflexion X1kg  log10Value     0.980 0.586         ns      
##  8 rotation       X1kg  log10Value     0.765 0.000000285   ****    
##  9 extension      X2kg  log10Value     0.965 0.174         ns      
## 10 flexion        X2kg  log10Value     0.962 0.127         ns      
## 11 lateralflexion X2kg  log10Value     0.970 0.258         ns      
## 12 rotation       X2kg  log10Value     0.628 0.00000000114 ****    
## 13 extension      X3kg  log10Value     0.973 0.330         ns      
## 14 flexion        X3kg  log10Value     0.877 0.000143      ***     
## 15 lateralflexion X3kg  log10Value     0.980 0.583         ns      
## 16 rotation       X3kg  log10Value     0.946 0.0294        *
```

-> similar results as raw values, for consistency reasons and easier to handle data, all statistics is performed on log-transformed data

## Perform Statistics

Use a generalized linear mixed model using Proband as random factor (blocked for Proband).

First, we need to decide which model more accurately represents the data:

- Additive model: ~ Load + Type -> no interaction between the two independent fixed effects
- Multiplicative model: ~Load\*Type -> model assumes an interaction between the two fixed effects

Perform maximum-likelihood testing (MLT) to test the two possible models:

- if p-value < 0.05 and lower BIC and AIC, choose model with more degree of freedom (multiplicative model)
- otherwise choose the less complex model (additive model) without interaction-effect

Intercept at Type==“extension” and Load==“0kg”

```
data$Type <- as.factor(data$Type)
data$Type <-  relevel(data$Type, ref = "extension")

##Define multiplicative Linear Mixed Model
full.model <- lmer(log10Value ~ Load * Type +  (1|Proband), data)
summary(full.model)
```

```
## Linear mixed model fit by REML. t-tests use Satterthwaite's method [
## lmerModLmerTest]
## Formula: log10Value ~ Load * Type + (1 | Proband)
##    Data: data
## 
## REML criterion at convergence: -1773.3
## 
## Scaled residuals: 
##     Min      1Q  Median      3Q     Max 
## -8.6587 -0.4876  0.0632  0.5819  2.4734 
## 
## Random effects:
##  Groups   Name        Variance Std.Dev.
##  Proband  (Intercept) 0.002500 0.05000 
##  Residual             0.004176 0.06462 
## Number of obs: 752, groups:  Proband, 47
## 
## Fixed effects:
##                               Estimate Std. Error         df t value Pr(>|t|)
## (Intercept)                   1.810289   0.011918 237.181604 151.897  < 2e-16
## LoadX1kg                     -0.078337   0.013330 690.000000  -5.877 6.52e-09
## LoadX2kg                     -0.067248   0.013330 690.000000  -5.045 5.81e-07
## LoadX3kg                     -0.063710   0.013330 690.000000  -4.779 2.15e-06
## Typeflexion                  -0.003473   0.013330 690.000000  -0.260 0.794557
## Typelateralflexion           -0.185854   0.013330 690.000000 -13.942  < 2e-16
## Typerotation                  0.065419   0.013330 690.000000   4.907 1.15e-06
## LoadX1kg:Typeflexion          0.103910   0.018852 690.000000   5.512 5.02e-08
## LoadX2kg:Typeflexion          0.095813   0.018852 690.000000   5.082 4.81e-07
## LoadX3kg:Typeflexion          0.063237   0.018852 690.000000   3.354 0.000839
## LoadX1kg:Typelateralflexion   0.080322   0.018852 690.000000   4.261 2.32e-05
## LoadX2kg:Typelateralflexion   0.088552   0.018852 690.000000   4.697 3.18e-06
## LoadX3kg:Typelateralflexion   0.104914   0.018852 690.000000   5.565 3.75e-08
## LoadX1kg:Typerotation         0.054167   0.018852 690.000000   2.873 0.004188
## LoadX2kg:Typerotation         0.037449   0.018852 690.000000   1.986 0.047376
## LoadX3kg:Typerotation         0.043361   0.018852 690.000000   2.300 0.021743
##                                
## (Intercept)                 ***
## LoadX1kg                    ***
## LoadX2kg                    ***
## LoadX3kg                    ***
## Typeflexion                    
## Typelateralflexion          ***
## Typerotation                ***
## LoadX1kg:Typeflexion        ***
## LoadX2kg:Typeflexion        ***
## LoadX3kg:Typeflexion        ***
## LoadX1kg:Typelateralflexion ***
## LoadX2kg:Typelateralflexion ***
## LoadX3kg:Typelateralflexion ***
## LoadX1kg:Typerotation       ** 
## LoadX2kg:Typerotation       *  
## LoadX3kg:Typerotation       *  
## ---
## Signif. codes:  0 '***' 0.001 '**' 0.01 '*' 0.05 '.' 0.1 ' ' 1
```

```
## 
## Correlation matrix not shown by default, as p = 16 > 12.
## Use print(x, correlation=TRUE)  or
##     vcov(x)        if you need it
```

```
##Define additive Linear Mixed Model
red.model <- lmer(log10Value ~  Load + Type  +  (1|Proband), data)
summary(red.model)
```

```
## Linear mixed model fit by REML. t-tests use Satterthwaite's method [
## lmerModLmerTest]
## Formula: log10Value ~ Load + Type + (1 | Proband)
##    Data: data
## 
## REML criterion at convergence: -1773.3
## 
## Scaled residuals: 
##     Min      1Q  Median      3Q     Max 
## -8.4973 -0.5106  0.0643  0.6420  2.6262 
## 
## Random effects:
##  Groups   Name        Variance Std.Dev.
##  Proband  (Intercept) 0.002480 0.04980 
##  Residual             0.004487 0.06698 
## Number of obs: 752, groups:  Proband, 47
## 
## Fixed effects:
##                      Estimate Std. Error         df t value Pr(>|t|)    
## (Intercept)          1.768306   0.009723 116.312153 181.871  < 2e-16 ***
## LoadX1kg            -0.018738   0.006909 699.000000  -2.712  0.00685 ** 
## LoadX2kg            -0.011795   0.006909 699.000000  -1.707  0.08822 .  
## LoadX3kg            -0.010832   0.006909 699.000000  -1.568  0.11736    
## Typeflexion          0.062267   0.006909 699.000000   9.013  < 2e-16 ***
## Typelateralflexion  -0.117407   0.006909 699.000000 -16.994  < 2e-16 ***
## Typerotation         0.099163   0.006909 699.000000  14.353  < 2e-16 ***
## ---
## Signif. codes:  0 '***' 0.001 '**' 0.01 '*' 0.05 '.' 0.1 ' ' 1
## 
## Correlation of Fixed Effects:
##             (Intr) LdX1kg LdX2kg LdX3kg Typflx Typltr
## LoadX1kg    -0.355                                   
## LoadX2kg    -0.355  0.500                            
## LoadX3kg    -0.355  0.500  0.500                     
## Typeflexion -0.355  0.000  0.000  0.000              
## Typltrlflxn -0.355  0.000  0.000  0.000  0.500       
## Typerotatin -0.355  0.000  0.000  0.000  0.500  0.500
```

```
##Perform maximum-likelihood testing 
anova(full.model, red.model)
```

```
## refitting model(s) with ML (instead of REML)
```

```
## Data: data
## Models:
## red.model: log10Value ~ Load + Type + (1 | Proband)
## full.model: log10Value ~ Load * Type + (1 | Proband)
##            npar     AIC     BIC logLik deviance  Chisq Df Pr(>Chisq)    
## red.model     9 -1813.3 -1771.7 915.64  -1831.3                         
## full.model   18 -1855.0 -1771.8 945.49  -1891.0 59.714  9  1.522e-09 ***
## ---
## Signif. codes:  0 '***' 0.001 '**' 0.01 '*' 0.05 '.' 0.1 ' ' 1
```

–> here maximum-likelihood testing suggests that a full multiplicative model involving an interaction-term captures the effect of the variables better:

- p-value < 0.05 and lower AIC and BIC

–> we proceed further analysis with the full model which allows us to perform post-hoc tests contrasting different groups (unlike an additive model)

## Plot Model Predictions and Main-Effects of Load and Type

```
## Overall Global Effects of Load and Type using Type III Analysis of Variance Table with Satterthwaite's method
anova(full.model)
```

```
## Type III Analysis of Variance Table with Satterthwaite's method
##           Sum Sq Mean Sq NumDF DenDF  F value    Pr(>F)    
## Load      0.0338 0.01127     3   690   2.6981   0.04493 *  
## Type      5.0779 1.69265     3   690 405.3276 < 2.2e-16 ***
## Load:Type 0.2547 0.02830     9   690   6.7767 2.346e-09 ***
## ---
## Signif. codes:  0 '***' 0.001 '**' 0.01 '*' 0.05 '.' 0.1 ' ' 1
```

```
emmip(full.model, Type ~ Load)
```

```
df <- summary(full.model)
df <- as.data.frame(df$coefficients)
df$`FoldChange [%]` <- 100*(10^df$Estimate - 1)
df <- signif(df, digits = 4)
df <- add_significance(df, p.col="Pr(>|t|)")
DT::datatable(df)
```

## Perform Post-Hoc Analysis

```
emTest <- emmeans(full.model, pairwise ~ Load | Type)
emcontr <- as.data.frame(emTest$contrasts)
emcontr$`FoldChange [%]` <- 100*(10^-emcontr$estimate -1)

emcontr <- emcontr %>% 
 mutate_if(is.numeric, signif, digits=3) %>% add_significance(p.col="p.value")

DT::datatable(emcontr)
```

## Plotting ROM

# Joint Precision Error (JPE)

## Load Data and Prepare Tables

Load Raw data and log10-transform them

```
data <- data.frame(read_excel("./JPE-Axial Load on Cervical Spine.xlsx"))
data$Proband <- as.factor(data$Proband)
data[,c(2,3,4,5)] <- sapply(data[,c(2,3,4,5)], FUN=as.numeric)

#take out probands who had missing measurements (row-wise) with NaNs
data <- data[complete.cases(data),]
data <- melt(data, id.vars=c("Proband", "Type"), variable.name = "Load")
data <- aggregate(data, by=list(Type=data$Type, Proband=data$Proband, Load=data$Load), FUN=mean, na.rm=TRUE)
data <- data[, colSums(is.na(data)) != nrow(data)]

##Log transform raw values
data$log10Value <- log10(data$value)
DT::datatable(data)
```

## Summarize Table

```
dat <- data %>%
    group_by(Load, Type) %>%
    summarize(count = n(),
              Mean = mean(value, na.rm=TRUE),
              Standard.Deviation = sd(value, na.rm=TRUE),
              lower.CI = mean(value, na.rm=TRUE) - qt(1 - (0.05 / 2), n() - 1) * sd(value, na.rm=TRUE) / sqrt(n()),
              upper.CI = mean(value, na.rm=TRUE) + qt(1 - (0.05 / 2), n() - 1) * sd(value, na.rm=TRUE) / sqrt(n())) %>% mutate_if(is.numeric, signif, digits=4)
```

```
## `summarise()` has grouped output by 'Load'. You can override using the `.groups` argument.
```

```
## `mutate_if()` ignored the following grouping variables:
## Column `Load`
```

```
DT::datatable(dat)
```

## Normality Testing

First with all values together: - create QQ-Plots, histograms and perform a Shapiro-Wilk Normality Test (p<0.05 is non-normally distributed)

```
p1 <- ggqqplot(data$value) + labs(title="QQ-Plot of all Raw Values")
p2 <- ggqqplot(data$log10Value) + labs(title="QQ-Plot of all log-transformed Values")

p3 <- ggdensity(data$value)+ labs(title="Histogram of all Raw Values")
p4 <- ggdensity(data$log10Value)+ labs(title="Histogram of log-transformed Values")

ggarrange(p1, p2, p3, p4,
          ncol = 2, nrow = 2)
```

```
## Raw Values
shapiro.test(data$value)
```

```
## 
##  Shapiro-Wilk normality test
## 
## data:  data$value
## W = 0.75039, p-value < 2.2e-16
```

```
## Log10-Transformed Values
shapiro.test(data$log10Value)
```

```
## 
##  Shapiro-Wilk normality test
## 
## data:  data$log10Value
## W = 0.97937, p-value = 5.249e-09
```

Now split according to groups (Load ~ Type) and plot raw values and perform Shapiro-Wilk Normality Tests (p<0.05 is non-normally distributed)

```
ggqqplot(data, "value", ggtheme = theme_bw()) +
  facet_grid(Load ~ Type, labeller = "label_both") + labs(title="QQ-Plots of group-splitted raw values")
```

```
ggdensity(data , "value") +
  facet_grid(Load ~ Type, labeller = "label_both") + labs(title="Histograms of group-splitted raw values")
```

```
data$rawVal <- as.numeric(data$value)
data$variable <- NULL
data %>%
  group_by(Load, Type) %>%
  shapiro_test(rawVal) %>%
 add_significance("p")
```

```
## # A tibble: 16 x 6
##    Type           Load  variable statistic             p p.signif
##    <chr>          <fct> <chr>        <dbl>         <dbl> <chr>   
##  1 extension      X0kg  rawVal       0.741 0.0000000747  ****    
##  2 flexion        X0kg  rawVal       0.641 0.00000000106 ****    
##  3 lateralflexion X0kg  rawVal       0.774 0.000000356   ****    
##  4 rotation       X0kg  rawVal       0.912 0.00137       **      
##  5 extension      X1kg  rawVal       0.770 0.000000287   ****    
##  6 flexion        X1kg  rawVal       0.768 0.000000217   ****    
##  7 lateralflexion X1kg  rawVal       0.735 0.0000000585  ****    
##  8 rotation       X1kg  rawVal       0.925 0.00402       **      
##  9 extension      X2kg  rawVal       0.844 0.0000149     ****    
## 10 flexion        X2kg  rawVal       0.844 0.0000124     ****    
## 11 lateralflexion X2kg  rawVal       0.891 0.000337      ***     
## 12 rotation       X2kg  rawVal       0.783 0.000000457   ****    
## 13 extension      X3kg  rawVal       0.901 0.000692      ***     
## 14 flexion        X3kg  rawVal       0.878 0.000116      ***     
## 15 lateralflexion X3kg  rawVal       0.912 0.00157       **      
## 16 rotation       X3kg  rawVal       0.942 0.0184        *
```

Now split according to groups (Load ~ Type) and plot log-transformed values

```
ggqqplot(data, "log10Value", ggtheme = theme_bw()) +
  facet_grid(Load ~ Type, labeller = "label_both") + labs(title="QQ-Plots of group-splitted log-transformed values")
```

```
ggdensity(data , "log10Value") +
  facet_grid(Load ~ Type, labeller = "label_both") + labs(title="Histogram of group-splitted log-transformed values")
```

```
data %>%
  group_by(Load, Type) %>%
  shapiro_test(log10Value) %>%
 add_significance("p")
```

```
## # A tibble: 16 x 6
##    Type           Load  variable   statistic       p p.signif
##    <chr>          <fct> <chr>          <dbl>   <dbl> <chr>   
##  1 extension      X0kg  log10Value     0.959 0.0892  ns      
##  2 flexion        X0kg  log10Value     0.932 0.00754 **      
##  3 lateralflexion X0kg  log10Value     0.953 0.0532  ns      
##  4 rotation       X0kg  log10Value     0.982 0.670   ns      
##  5 extension      X1kg  log10Value     0.988 0.899   ns      
##  6 flexion        X1kg  log10Value     0.987 0.871   ns      
##  7 lateralflexion X1kg  log10Value     0.965 0.166   ns      
##  8 rotation       X1kg  log10Value     0.990 0.951   ns      
##  9 extension      X2kg  log10Value     0.970 0.249   ns      
## 10 flexion        X2kg  log10Value     0.974 0.340   ns      
## 11 lateralflexion X2kg  log10Value     0.949 0.0372  *       
## 12 rotation       X2kg  log10Value     0.966 0.164   ns      
## 13 extension      X3kg  log10Value     0.983 0.704   ns      
## 14 flexion        X3kg  log10Value     0.977 0.451   ns      
## 15 lateralflexion X3kg  log10Value     0.978 0.517   ns      
## 16 rotation       X3kg  log10Value     0.985 0.789   ns
```

## Perform Statistics

Use a generalized linear mixed model using Proband as random factor (blocked for Proband).

First, we need to decide which model more accurately represents the data:

- Additive model: ~ Load + Type -> no interaction between the two independent fixed effects
- Multiplicative model: ~Load\*Type -> model assumes an interaction between the two fixed effects

Perform maximum-likelihood testing (MLT) to test the two possible models:

- if p-value < 0.05 and lower BIC and AIC, choose model with more degree of freedom (multiplicative model)
- otherwise choose the less complex model (additive model) without interaction-effect

Intercept at Type==“extension” and Load==“0kg”

```
data$Type <- as.factor(data$Type)
data$Type <-  relevel(data$Type, ref = "extension")

##Define multiplicative Linear Mixed Model
full.model <- lmer(log10Value ~ Load * Type +  (1|Proband), data)
summary(full.model)
```

```
## Linear mixed model fit by REML. t-tests use Satterthwaite's method [
## lmerModLmerTest]
## Formula: log10Value ~ Load * Type + (1 | Proband)
##    Data: data
## 
## REML criterion at convergence: 90.9
## 
## Scaled residuals: 
##     Min      1Q  Median      3Q     Max 
## -3.3407 -0.5986 -0.0303  0.5721  3.0833 
## 
## Random effects:
##  Groups   Name        Variance Std.Dev.
##  Proband  (Intercept) 0.01761  0.1327  
##  Residual             0.05423  0.2329  
## Number of obs: 776, groups:  Proband, 49
## 
## Fixed effects:
##                               Estimate Std. Error         df t value Pr(>|t|)
## (Intercept)                   0.784289   0.038616 408.247582  20.310  < 2e-16
## LoadX1kg                      0.038151   0.047536 711.871080   0.803    0.422
## LoadX2kg                      0.055935   0.047536 711.871080   1.177    0.240
## LoadX3kg                      0.028584   0.047536 711.871080   0.601    0.548
## Typeflexion                   0.032361   0.047315 712.311488   0.684    0.494
## Typelateralflexion            0.034536   0.047536 711.871080   0.727    0.468
## Typerotation                 -0.202774   0.047315 712.311488  -4.286 2.07e-05
## LoadX1kg:Typeflexion          0.026975   0.066883 711.871080   0.403    0.687
## LoadX2kg:Typeflexion          0.022826   0.066883 711.871080   0.341    0.733
## LoadX3kg:Typeflexion          0.033317   0.066883 711.871080   0.498    0.619
## LoadX1kg:Typelateralflexion  -0.052227   0.067227 711.871080  -0.777    0.437
## LoadX2kg:Typelateralflexion  -0.025906   0.067227 711.871080  -0.385    0.700
## LoadX3kg:Typelateralflexion   0.006803   0.067227 711.871080   0.101    0.919
## LoadX1kg:Typerotation        -0.022383   0.066883 711.871080  -0.335    0.738
## LoadX2kg:Typerotation        -0.019172   0.066883 711.871080  -0.287    0.774
## LoadX3kg:Typerotation        -0.027914   0.066883 711.871080  -0.417    0.677
##                                
## (Intercept)                 ***
## LoadX1kg                       
## LoadX2kg                       
## LoadX3kg                       
## Typeflexion                    
## Typelateralflexion             
## Typerotation                ***
## LoadX1kg:Typeflexion           
## LoadX2kg:Typeflexion           
## LoadX3kg:Typeflexion           
## LoadX1kg:Typelateralflexion    
## LoadX2kg:Typelateralflexion    
## LoadX3kg:Typelateralflexion    
## LoadX1kg:Typerotation          
## LoadX2kg:Typerotation          
## LoadX3kg:Typerotation          
## ---
## Signif. codes:  0 '***' 0.001 '**' 0.01 '*' 0.05 '.' 0.1 ' ' 1
```

```
## 
## Correlation matrix not shown by default, as p = 16 > 12.
## Use print(x, correlation=TRUE)  or
##     vcov(x)        if you need it
```

```
##Define additive Linear Mixed Model
red.model <- lmer(log10Value ~  Load + Type  +  (1|Proband), data)
summary(red.model)
```

```
## Linear mixed model fit by REML. t-tests use Satterthwaite's method [
## lmerModLmerTest]
## Formula: log10Value ~ Load + Type + (1 | Proband)
##    Data: data
## 
## REML criterion at convergence: 57
## 
## Scaled residuals: 
##     Min      1Q  Median      3Q     Max 
## -3.2940 -0.6029 -0.0301  0.5789  3.0205 
## 
## Random effects:
##  Groups   Name        Variance Std.Dev.
##  Proband  (Intercept) 0.01764  0.1328  
##  Residual             0.05374  0.2318  
## Number of obs: 776, groups:  Proband, 49
## 
## Fixed effects:
##                     Estimate Std. Error        df t value Pr(>|t|)    
## (Intercept)          0.78785    0.02915 175.55606  27.028   <2e-16 ***
## LoadX1kg             0.02639    0.02354 720.87129   1.121   0.2626    
## LoadX2kg             0.05045    0.02354 720.87129   2.143   0.0324 *  
## LoadX3kg             0.03163    0.02354 720.87129   1.344   0.1794    
## Typeflexion          0.05313    0.02358 722.62852   2.253   0.0246 *  
## Typelateralflexion   0.01670    0.02366 720.87129   0.706   0.4804    
## Typerotation        -0.22015    0.02358 722.62852  -9.335   <2e-16 ***
## ---
## Signif. codes:  0 '***' 0.001 '**' 0.01 '*' 0.05 '.' 0.1 ' ' 1
## 
## Correlation of Fixed Effects:
##             (Intr) LdX1kg LdX2kg LdX3kg Typflx Typltr
## LoadX1kg    -0.404                                   
## LoadX2kg    -0.404  0.500                            
## LoadX3kg    -0.404  0.500  0.500                     
## Typeflexion -0.410  0.000  0.000  0.000              
## Typltrlflxn -0.406  0.000  0.000  0.000  0.502       
## Typerotatin -0.410  0.000  0.000  0.000  0.507  0.502
```

```
##Perform maximum-likelihood testing 
anova(full.model, red.model)
```

```
## refitting model(s) with ML (instead of REML)
```

```
## Data: data
## Models:
## red.model: log10Value ~ Load + Type + (1 | Proband)
## full.model: log10Value ~ Load * Type + (1 | Proband)
##            npar    AIC     BIC  logLik deviance  Chisq Df Pr(>Chisq)
## red.model     9 33.695  75.582 -7.8475   15.695                     
## full.model   18 49.211 132.985 -6.6053   13.211 2.4843  9     0.9813
```

-> p-value > 0.05 and high BIC and AIC does not justify the use of a more complex model with interaction-effect

-> we will continue with the additive model and look at the independent effects of load and type of movement

-> this model does not allow us to contrast for spefici load effects within different type of movements (unlike ROM), since our model does not include an interaction-term

## Plot Model Predictions and Main-Effects of Load and Type

```
## Overall Global Effects of Load and Type using Type III Analysis of Variance Table with Satterthwaite's method
anova(red.model)
```

```
## Type III Analysis of Variance Table with Satterthwaite's method
##      Sum Sq Mean Sq NumDF  DenDF F value Pr(>F)    
## Load 0.2523 0.08411     3 720.87  1.5651 0.1965    
## Type 8.9705 2.99015     3 722.04 55.6420 <2e-16 ***
## ---
## Signif. codes:  0 '***' 0.001 '**' 0.01 '*' 0.05 '.' 0.1 ' ' 1
```

```
emmip(red.model, Type ~ Load)
```

```
df <- summary(red.model)
df <- as.data.frame(df$coefficients)
df$`FoldChange [%]` <- 100*(10^df$Estimate - 1)

df <- signif(df, digits = 4)
df <- add_significance(df, p.col="Pr(>|t|)")
DT::datatable(df)
```

-> fold-change compared to intercept (Type== “Extention” / Load == “0kg”)

## Plot Data

### Session Information

```
sessionInfo()
```

```
## R version 4.0.3 (2020-10-10)
## Platform: x86_64-pc-linux-gnu (64-bit)
## Running under: Ubuntu 18.04 LTS
## 
## Matrix products: default
## BLAS:   /usr/lib/x86_64-linux-gnu/openblas/libblas.so.3
## LAPACK: /usr/lib/x86_64-linux-gnu/libopenblasp-r0.2.20.so
## 
## locale:
##  [1] LC_CTYPE=C.UTF-8       LC_NUMERIC=C           LC_TIME=C.UTF-8       
##  [4] LC_COLLATE=C.UTF-8     LC_MONETARY=C.UTF-8    LC_MESSAGES=C.UTF-8   
##  [7] LC_PAPER=C.UTF-8       LC_NAME=C              LC_ADDRESS=C          
## [10] LC_TELEPHONE=C         LC_MEASUREMENT=C.UTF-8 LC_IDENTIFICATION=C   
## 
## attached base packages:
## [1] stats     graphics  grDevices utils     datasets  methods   base     
## 
## other attached packages:
##  [1] rstatix_0.7.0   ggpubr_0.4.0    dplyr_1.0.5     DT_0.18        
##  [5] ggplot2_3.3.4   emmeans_1.6.2-1 reshape2_1.4.4  lmerTest_3.1-3 
##  [9] lme4_1.1-26     Matrix_1.3-4    readxl_1.3.1   
## 
## loaded via a namespace (and not attached):
##  [1] sass_0.4.0          tidyr_1.1.3         jsonlite_1.7.2     
##  [4] splines_4.0.3       carData_3.0-4       bslib_0.2.5.1      
##  [7] assertthat_0.2.1    statmod_1.4.36      highr_0.9          
## [10] cellranger_1.1.0    yaml_2.2.1          numDeriv_2016.8-1.1
## [13] pillar_1.5.1        backports_1.2.1     lattice_0.20-44    
## [16] glue_1.4.2          digest_0.6.27       ggsignif_0.6.1     
## [19] minqa_1.2.4         colorspace_2.0-2    sandwich_3.0-1     
## [22] cowplot_1.1.1       htmltools_0.5.1.1   plyr_1.8.6         
## [25] pkgconfig_2.0.3     broom_0.7.5         haven_2.3.1        
## [28] purrr_0.3.4         xtable_1.8-4        mvtnorm_1.1-2      
## [31] scales_1.1.1        openxlsx_4.2.4      rio_0.5.26         
## [34] tibble_3.1.0        farver_2.1.0        generics_0.1.0     
## [37] car_3.0-10          ellipsis_0.3.2      TH.data_1.0-10     
## [40] withr_2.4.2         pbkrtest_0.5.1      cli_3.0.1          
## [43] survival_3.2-10     magrittr_2.0.1      crayon_1.4.1       
## [46] estimability_1.3    evaluate_0.14       fansi_0.5.0        
## [49] nlme_3.1-152        MASS_7.3-54         forcats_0.5.1      
## [52] foreign_0.8-81      tools_4.0.3         data.table_1.14.0  
## [55] hms_1.1.0           lifecycle_1.0.0     multcomp_1.4-16    
## [58] stringr_1.4.0       munsell_0.5.0       zip_2.2.0          
## [61] compiler_4.0.3      jquerylib_0.1.4     rlang_0.4.11       
## [64] grid_4.0.3          nloptr_1.2.2.2      rstudioapi_0.13    
## [67] htmlwidgets_1.5.3   crosstalk_1.1.1     labeling_0.4.2     
## [70] rmarkdown_2.7       boot_1.3-28         gtable_0.3.0       
## [73] codetools_0.2-18    abind_1.4-5         DBI_1.1.1          
## [76] curl_4.3.2          R6_2.5.0            zoo_1.8-9          
## [79] knitr_1.31          utf8_1.2.2          stringi_1.7.3      
## [82] parallel_4.0.3      Rcpp_1.0.7          vctrs_0.3.8        
## [85] tidyselect_1.1.0    xfun_0.24           coda_0.19-4
```
